# Endocytosis and Endocytic Neurotrophin Signaling in Developing Spiral Ganglion Neurons

**DOI:** 10.3390/ijms27198527

**Published:** 2026-09-24

**Authors:** Shannon Doolittle, Kwang Pak, Eduardo Chavez, Gary D. Housley, Arwa Kurabi, Allen F. Ryan

**Affiliations:** 1 Department of Otolaryngology, University of California San Diego (UCSD), La Jolla, CA 92093, USA; shandoolittle@gmail.com (S.D.); kpak@vapop.ucsd.edu (K.P.); edchavez@ucsd.edu (E.C.); akurabi@ucsd.edu (A.K.); 2 Audiology, Mary Bridge Children’s Hospital, Tacoma, WA 98405, USA; 3 Research Department, VA San Diego Medical Center, San Diego, CA 92161, USA; 4 Translational Neuroscience Facility, Department of Physiology, School of Biomedical Sciences, UNSW Sydney, Sydney, NSW 2052, Australia; g.housley@unsw.edu.au; 5 Department of Neurosciences, University of California San Diego (UCSD), La Jolla, CA 92093, USA

**Keywords:** spiral ganglion neurons, organotypic culture, neurotrophins, neuritogenesis, endocytosis

## Abstract

Spiral ganglion neurons (SGNs) relay auditory information from cochlear sensory cells to the central nervous system (CNS). During development, neurotrophins (NTs) secreted by sensory and supporting cells promote SGN dendrite outgrowth. In injury models, exogenous NTs have been used to stimulate SGN regeneration after damage. Following ligand binding, NT-Trk receptor complexes are internalized and may undergo recycling or degradation, or remain intracellular as “signaling endosomes” that can be retrogradely transported to the soma to influence gene expression, survival, and neuritogenesis. Here, we investigated the role of Trk-containing endosomes in SGNs. Neonatal rat SG explants were treated with BDNF and NT-3 in combination with inhibitors targeting distinct steps of endocytosis. Neurite number and length were quantified and compared to untreated controls. Inhibition of actin polymerization (cytochalasin-B) or dynamin (Dynasore) strongly suppressed neurite outgrowth in both NT-treated and untreated explants. Similarly, inhibitors of clathrin-mediated endocytosis, including those acting upstream of dynamin, reduced neuritogenesis under both conditions. In contrast, inhibition of clathrin-independent pathways diminished neurite growth only in the absence of NTs. Blocking retrograde transport of endosomes to the trans-Golgi network (Retro-2) selectively increased neurite length in NT-treated explants, while inhibition of retrograde transport along neurites (Mycalolide B) markedly reduced both neurite number and extension regardless of NT treatment. These findings suggest that basal SGN neuritogenesis relies on both clathrin-dependent and clathrin-independent endocytosis, whereas NT-induced neurite growth is specifically mediated through clathrin- and dynamin-dependent mechanisms. Although some inhibitors also disrupt actin polymerization, the lack of effect from clathrin-independent inhibitors supports clathrin-mediated internalization as the predominant pathway for NT-driven SGN growth.

## 1. Introduction

The neurons of the cochlear spiral ganglion (SG) transmit auditory information from cochlear sensory hair cells (HCs) to the central auditory system, making them essential for cochlear function and the perception of hearing. During development, the growth of SG neuron (SGN) afferent dendrites toward the HCs is supported by the neurotrophic growth factors neurotrophin-3 (NT-3) and brain-derived neurotrophic factor (BDNF) (e.g., [1,2]). These factors also differentially influence the functional characteristics of SG neurons, with NT-3 eliciting an apical-turn, low-frequency phenotype, while BDNF encourages a basal-turn, high-frequency phenotype [3]. Neurotrophins (NTs) have also been used to stimulate SGN regeneration after damage (e.g., [4]).

Neurotrophins interact with their cognate Trk receptors, TrkC for NT-3 and TrkB for BDNF, to enhance neurite extension. NTs induce Trk receptor dimerization and phosphorylation, which in turn activate downstream signaling cascades that mediate fiber growth and direction. This likely would include phospholipase C-dependent activation of TRPC channels, which in rat cerebellar granule cells provides tropism for growth cone migration [5].

After activation, Trk receptors are internalized into endosomes via endocytosis, which can occur by several mechanisms. There is strong evidence from other neural systems to support endocytosis mediated by clathrin and dynamin [6]. It is also well documented that ligand binding recruits Trk receptors into lipid rafts, which increases their activation [7] and potentially exposes them to clathrinid-independent caveolar endocytosis mediated by dynamin alone [8]. Finally, it has been found that macroendocytosis mediated by the pinocytotic chaperone Pincher, independent of both clathrin and caveolae, can mediate endocytosis of Trk receptors [9]. All of these processes involve surface-associated actin polymerization to mediate invagination of membrane pits.

Following internalization, the receptors can undergo a number of potential fates, summarized in Figure 1 [6]. They may be inactivated by fusion of endosomes with lysosomes. They may be recycled back to the membrane directly from endosomes. They may be retrogradely transported through the trans-Golgi network (as opposed to anterograde transport which occurs in de novo protein synthesis) into the Golgi apparatus, where they can be either degraded or released in exosomes for recycling to the membrane [10].

There is also increasing evidence that an alternative fate exists for endosomes containing both Trk receptors and bound NTs, known as “signaling endosomes”. These receptor/ligand-containing endosomes can influence events at the growth cone of the neurite. Alternatively, they can be transported to the cell body along the neurite by retrograde transport (Figure 2). This allows them to stimulate the cell nucleus, mediating gene expression and increasing neuron survival, as well as the synthesis of proteins required for neurite growth [6]. Inhibition of Trk activity in either the distal axons or the somata of cultured neurons reduces cell survival [11]. Furthermore, retrograde TrkB endosomal signaling, mediated by Ca^2+^, has been shown to support dendrite formation in cortical neurons [12].

However, signaling through PLC-γ, calcineurin, and dynamin1 limited to the distal process can promote neurite elongation independent of retrograde signaling and transcriptional responses [13].

The role of endocytosis in SG neuron stimulation by NTs has not been well studied. Kucharava et al. [14] concluded that hearing loss and cochlear neural abnormalities in mice deficient in NHE6 (sodium-hydrogen exchanger 6) are due to reduced BDNF/TrkB endosomal signaling, based on the putative role of NHE6 in early endosome development. However, endocytosis was not directly measured. Retrograde transport of signaling endosomes has been suggested as occurring in the cochlea [15], based primarily on its observation in other systems. Given the potential fates of Trk receptors, as described above, it is possible that receptor internalization serves primarily to limit the duration of receptor activation in SG neurons by rapid degradation. It is also possible that internalized receptors play a further role in NT activity, prior to their degradation, by one or more of the mechanisms discussed above.

In the cochlea, BDNF and NT-3 are well-established regulators of SGN survival, neurite growth, synaptic maintenance, and physiological phenotype, acting primarily through TrkB and TrkC receptors. Combined activation of TrkB and TrkC has also been shown to produce synergistic effects in postnatal SGNs. Our inhibitor data extend this framework by functionally testing whether endocytosis and retrograde endosomal trafficking are required for BDNF/NT-3-mediated SGN neuritogenesis [15]. To further investigate the roles and mechanisms of endocytosis in SGN neurotrophic signaling, we determined whether inhibitors of endocytosis, retrograde transport into Golgi, or retrograde transport along neurites altered the number and length of fibers extending from NT-treated SG explants in the presence of these neurotrophic factors.

## 2. Results

To investigate how endocytic trafficking regulates SGN neurotrophic signaling, neonatal rat SG explants were treated with BDNF and NT-3 in the presence or absence of inhibitors targeting distinct trafficking steps. Neurite number and length were quantified as measures of initiation and extension and compared across inhibitor-treated explants and untreated and neurotrophin-plus-DMSO-vehicle controls.

Control SG explants treated with DMSO carrier were included within each of the experiments to control for variability in animal/explant characteristics in the bioassay. Across all experiments, explant neurite length averaged 295.5 µm (± 111.2 µm, SEM = 17.6 µm), while the average control lengths for individual inhibitor experiments ranged from 263.3 to 328.0 µm. The number of neurites per explant averaged 23.4 overall (± 27.3, SEM = 4.5), while for the individual experiments, average control numbers ranged from 10.6 to 59.7. The control results for each experiment were compared to those observed after treatment with the tested inhibitor concentration for that experiment, as listed in Table in the methods and illustrated in Figure 3, Figure 4, Figure 5, Figure 6, Figure 7, Figure 8 and Figure 9.

Among the inhibitors tested, genistein and chlorpromazine produced the most pronounced effects. Explants treated with either inhibitor showed no detectable neurite extension or outgrowth. Other inhibitors produced partial or pathway-specific effects on neurite number and length, summarized below.

### 2.1. ES9-17

Endosidin 9 analog 17 (ES9-17) is a non-protonophoric clathrin-mediated endocytosis inhibitor and a ligand to the clathrin heavy chain (CHC), which makes up the length of each leg and associates with a clathrin light chain (CLC). ES9-17 binds directly to the N-terminal domain of the CHC with an EC_50_ of approximately 13–17 μM [16]. As illustrated in Figure 3, treatment with ES9-17 at 50 and 100 µM concentrations primarily reduced neurite initiation by reducing the number of neurites without significantly altering neurite length. Higher concentrations of ES9-17 (200 µM and 500 µM) nearly abolished neurotrophin-stimulated outgrowth.

**Figure 3:**
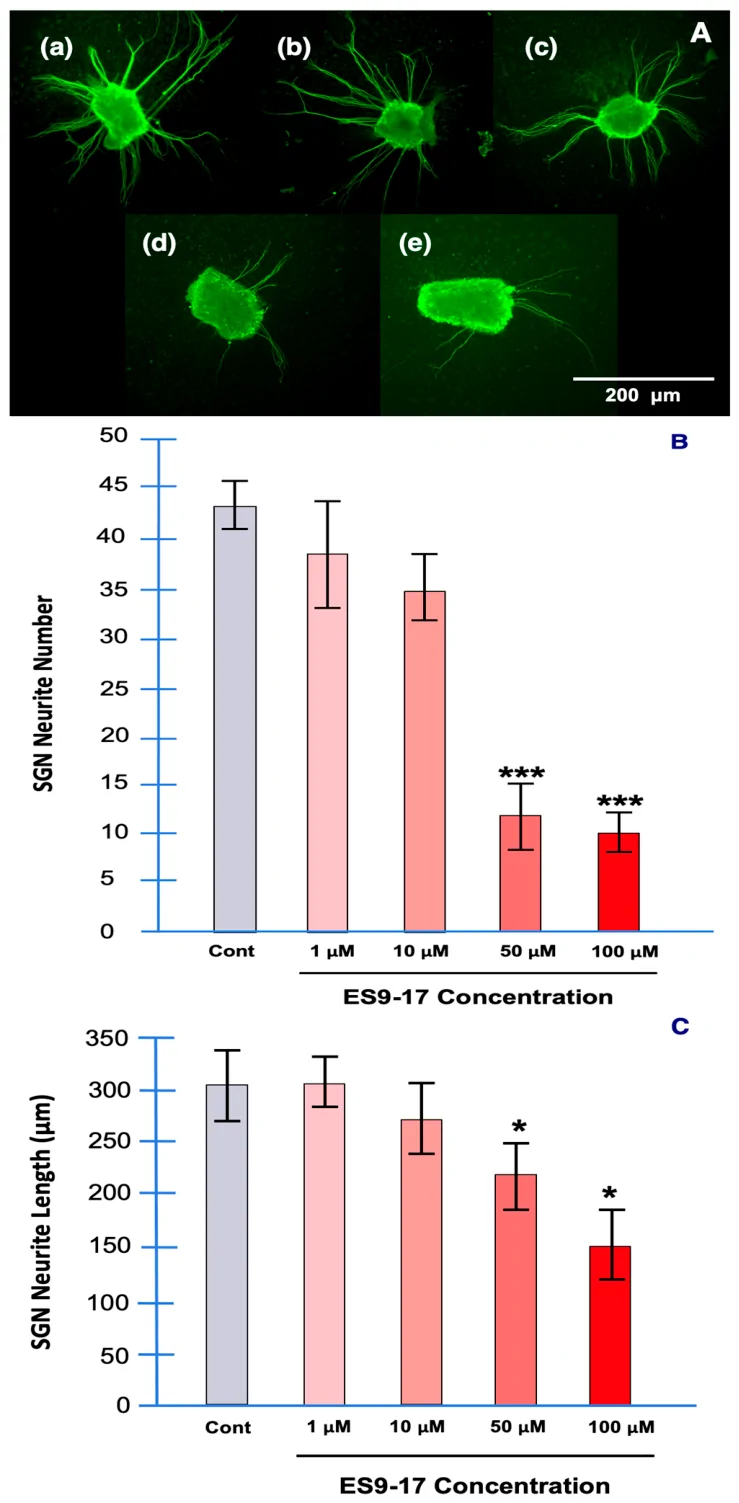
Effect of ES9-17, an inhibitor of clathrin-mediated endocytosis, on SGN neuritogenesis and neurite extension. From NF200 immunofluorescence imaging: (**A**) representative control explant (BDNF and NT-3 only, (**a**)) and explants treated with 1 μM, 10 μM, 50 μM or 100 μM of ES9-17 (**b**–**e**), in the presence of BDNF and NT-3. Average neurite number per explant (**B**) and average neurite length (**C**) were quantified in the presence of BDNF (10 ng/mL) and NT-3 (25 ng/mL), plus increasing concentrations of the inhibitor. Data are presented as mean ± standard error of the mean (SEM) from three independent experiments with N = 6–8 explants per condition. For this and subsequent figures, untreated explants without exogenous neurotrophins served as the negative control, and explants treated with BDNF and NT-3 without inhibitor served as the positive control. Cont = control (0.5% DMSO + media only); lines represent SEM; * = *p* < 0.05; *** = *p* < 0.001 (ANOVA).

### 2.2. Dynasore

Dynasore is a dynamin inhibitor that reduces clathrin-mediated endocytosis and most forms of clathrin-independent endocytosis, with an IC_50_ of ~15 µM [17]. As illustrated in Figure 4, treatment with Dynasore significantly decreased the number of neurites/SG explant by 80–90 percent compared to the control at 25 µM (ANOVA *p* < 0.01), 50 µM or 75 µM (ANOVA, *p* < 0.001) (Figure 4B), and decreased the average length of SG neurites by >90% at 50 µM or 75 µM (ANOVA, *p* < 0.01) (Figure 4C).

**Figure 4:**
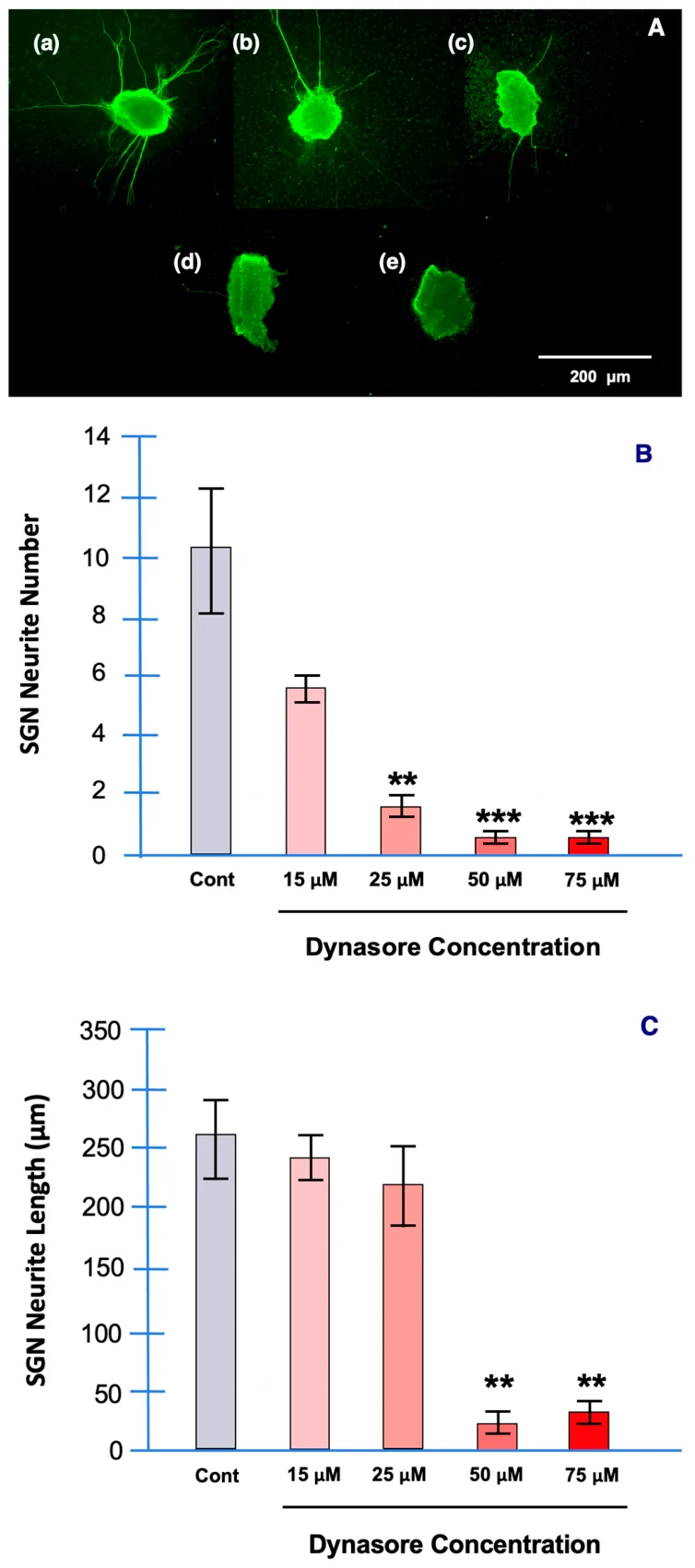
Effect of Dynasore, an inhibitor of both clathrin-mediated and several forms of clathrin-independent endocytosis, on NT-treated SGN neuritogenesis and extension. From NF200 immunofluorescence imaging: (**A**) representative control explant (BDNF and NT-3 only, (**a**)) and explants treated with 15 μM, 25 μM, 50 μM or 75 μM of Dynasore in the presence of BDNF and NT-3 (**b**–**e**). Average number (**B**) and average length (**C**) of neurites present on SG explants in the presence of BDNF (10 ng/mL) and NT-3 (25 ng/mL), plus increasing concentrations of the inhibitor. Control explants were cultured without exogenous neurotrophins as a negative control or treated with BDNF and NT-3 alone as a positive control. Each bar represents the average of nine explants from three independent replicates, with three explants in each replicate. ** = *p* < 0.01; *** = *p* < 0.001 (ANOVA).

### 2.3. Cytochalasin-B

Cytochalasin-B is a toxin that disrupts the formation of actin microfilaments by slowing down the “pulling” tension generated by growth cones to stop axons and dendrites from extending [18]. As illustrated in Figure 5, treatment with low-dose cytochalasin-B under BDNF/NT-3 conditions did not produce a reproducible biphasic enhancement of neurite growth; instead, increasing concentrations strongly suppressed neurite extension and initiation.

**Figure 5:**
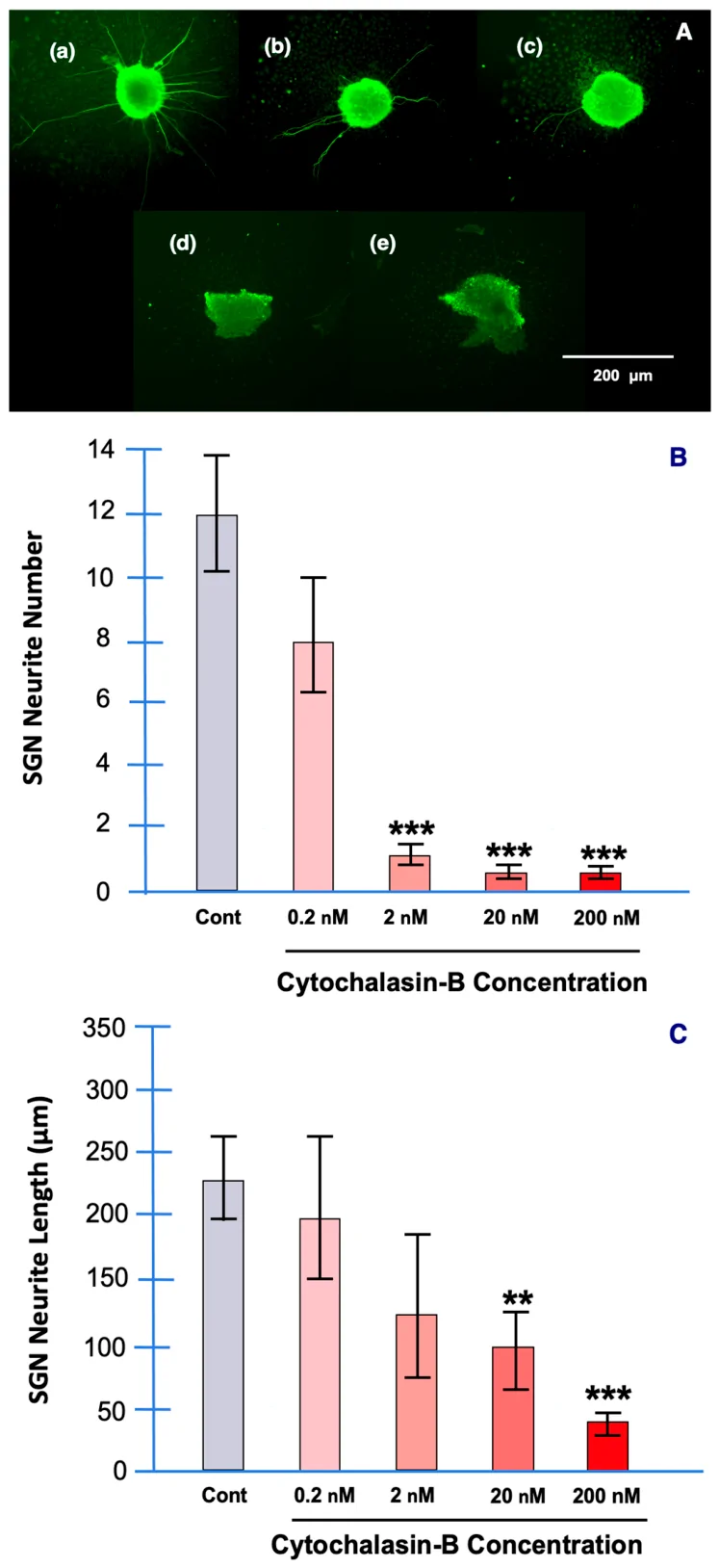
Effect of cytochalasin-B, an inhibitor of actin polymerization, on SGN neuritogenesis and neurite extension. Explants were treated with increasing concentrations of cytochalasin-B in the presence of BDNF and NT-3 to determine whether disruption of the actin filament network alters neurotrophin-stimulated neurite outgrowth. From NF200 immunofluorescence imaging: (**A**) representative control explant (BDNF and NT-3 only (**a**)) and explants treated with 0.2 nM, 2 nM, 20 nM or 200 nM (**b**–**e**) of cytochalasin-B, in the presence of BDNF and NT-3. Average neurite number per explant (**B**) and average neurite length (**C**) were quantified. Data are presented as mean ± SEM. Each bar represents the average of nine explants from three independent replicates, with three explants in each replicate. ** = *p* < 0.01; *** = *p* < 0.001 (ANOVA).

Together, these findings support clathrin/dynamin-mediated endocytosis as a major mechanism by which activated Trk receptors generate growth-promoting signaling endosomes in SGNs.

### 2.4. Pitstop 2

Pitstop 2 is a cell-permeable inhibitor of clathrin-mediated endocytosis with an IC_50_ of ~12 µM [19]. Figure 6 shows representative epifluorescence images of SG explants immunolabeled with anti-neurofilament 200 (green), showing neurite extension under control conditions and following co-treatment with Pitstop 2. Explants were cultured for 72 h in maintenance medium supplemented with BDNF and NT-3. Qualitative analysis of neurite outgrowth shown in Figure 6 reveals that treatment of explants with 25 µM Pitstop 2 decreased the number of neurites by >95% compared with control explants (ANOVA, *p* < 0.01) (Figure 6B) and reduced the average length of explant neurites by 45% at 10 μM and >90% at 25 μM (ANOVA, *p* < 0.01) (Figure 6C).

**Figure 6:**
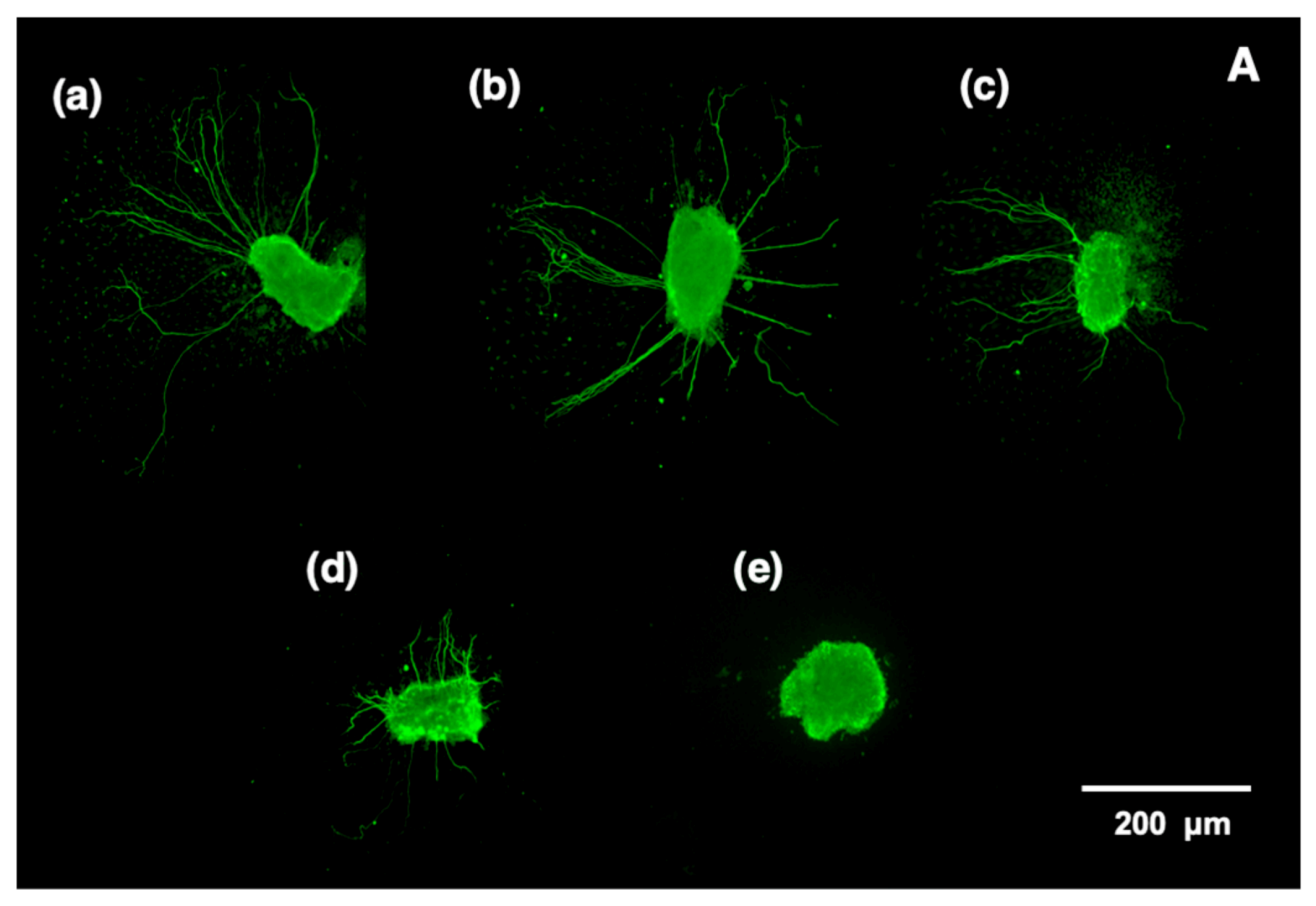

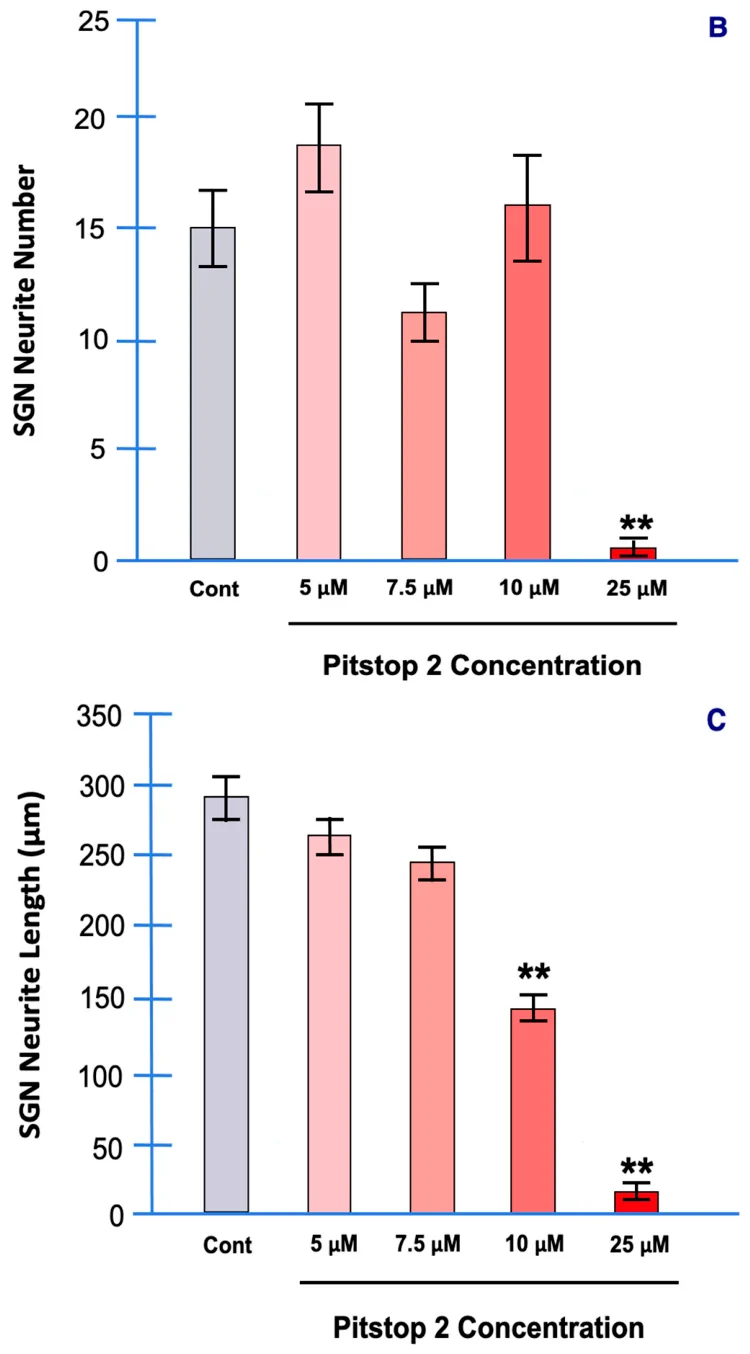
Effect of Pitstop 2, an inhibitor of clathrin-mediated endocytosis, on NT-treated SG explants. From NF200 immunofluorescence imaging: (**A**) representative control explant (**a**) and explants treated with 5 μM, 7.5 μM, 10 μM, or 25 μM of Pitstop 2 (**b**–**e**), in the presence of BDNF and NT-3. Average number (**B**) and average length (**C**) of neurites present on SG explants in the presence of BDNF and NT-3, as well as increasing concentrations of Pitstop 2 inhibitor, and relative to the untreated control. Each bar represents the average of nine explants from three independent replicates, with three explants in each replicate. ** = *p* < 0.01 (ANOVA).

### 2.5. Filipin

Filipin disrupts caveolae, and is an inhibitor of several forms of clathrin-independent endocytosis, with an IC_50_ of ~2 µM [20]. As shown in Figure 7B, Filipin had no statistically significant effect on neurite number per SG explant. While the average number of neurites was reduced at 25 µg/mL, the difference was not statistically significant. There was also no significant change in neurite length (Figure 7C).

**Figure 7:**
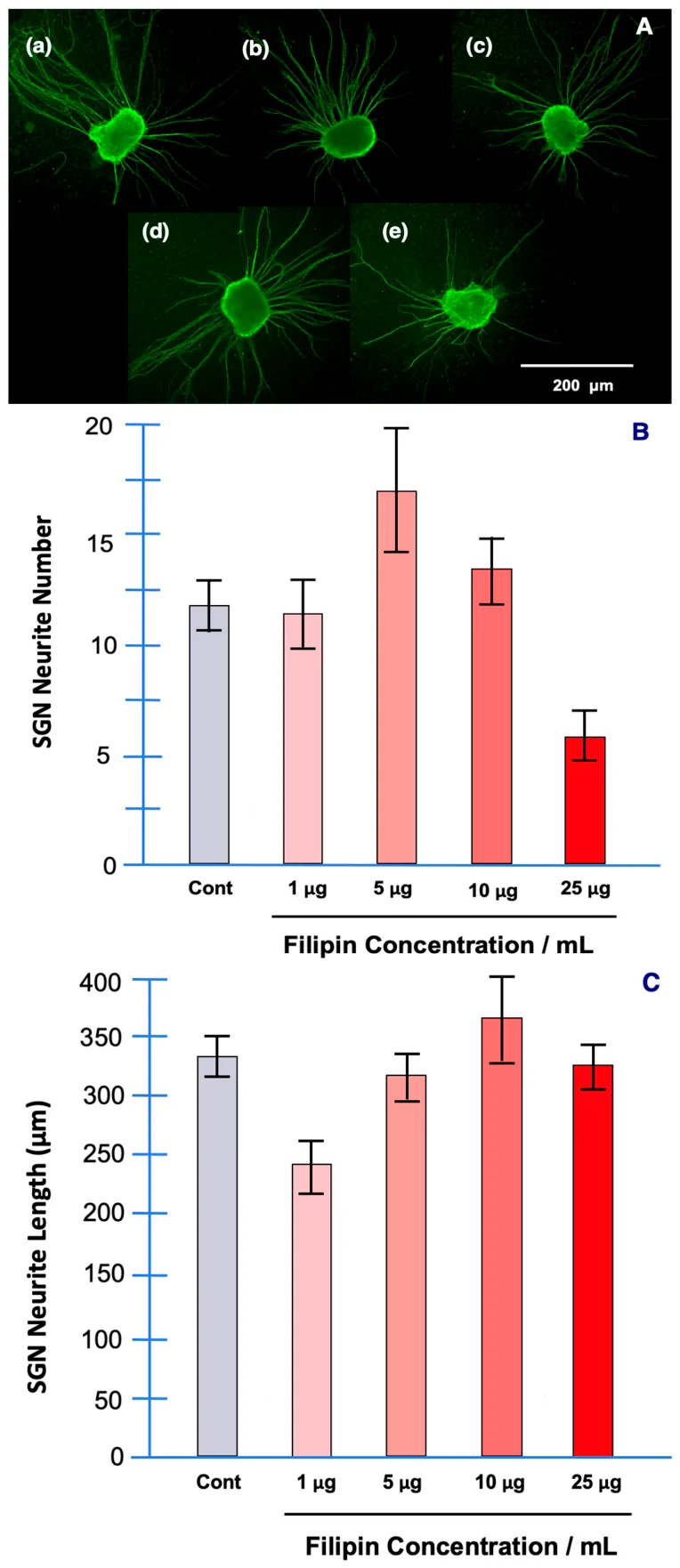
Lack of effect of Filipin, an inhibitor of clathrin-independent endocytosis, on SG explants neuritogenesis and extension. From NF200 immunofluorescence imaging: (**A**) representative control explant (BDNF and NT-3 only, (**a**)) and explants treated with 1 µg/mL, 5 µg/mL, 10 µg/mL or 25 µg/mL of Filipin, in the presence of BDNF and NT-3 (**b**–**e**). Average number (**B**) and average length (**C**) of neurites present on SG explants in the presence of BDNF and NT-3, as well as increasing concentrations of the inhibitor, and relative to the positive control treated with BDNF and NT-3 only. Each bar represents the average of nine explants from three independent replicates, with three explants in each replicate.

### 2.6. Retro-2

Retro-2 is an inhibitor of retrograde transport into the trans-Golgi network, with an IC_50_ of ~5 µM [21]. Retro-2 had no significant effect on explant neurite number at any dosage tested (Figure 8B). However, Retro-2 increased the length of neurites per SG explant by ~30 percent compared to control explants at 30 µM (ANOVA, *p* < 0.05) (Figure 8C).

**Figure 8:**
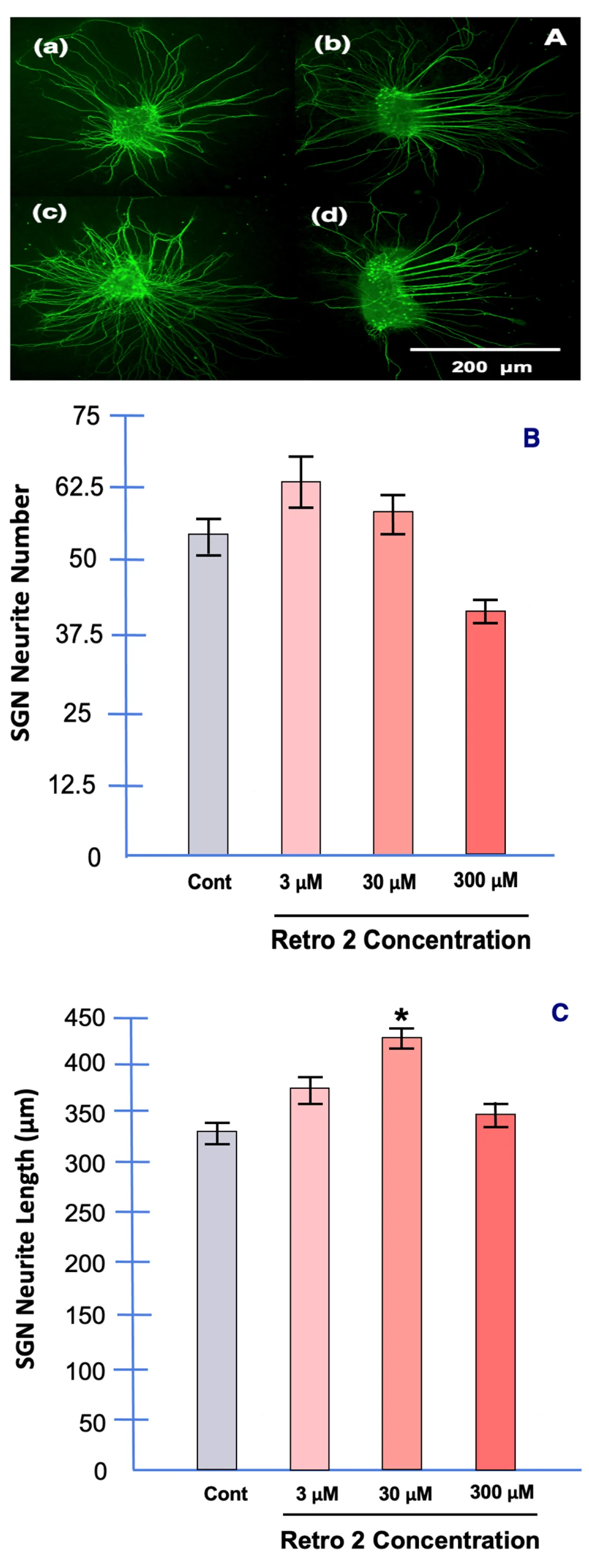
Effect of Retro-2, an inhibitor of retrograde transport into the trans-Golgi network, on SGN neuritogenesis and extension. From NF200 immunofluorescence imaging: (**A**) representative control explant (BDNF and NT-3 only, (**a**)) and explants treated with 3 µM, 30 µM or 300 µM of Retro-2, in the presence of BDNF and NT-3 (**b**–**d**). Average number (**B**) and average length (**C**) of neurites present on SG explants in the presence of BDNF and NT-3, as well as increasing concentrations of the inhibitor. Each bar represents the average of nine explants from three independent replicates, with three explants in each replicate. * = *p* < 0.05 (ANOVA).

### 2.7. Mycalolide B

The macrolide sponge toxin Mycalolide B dissociates dynactin and selectively inhibits retrograde transport axonal transport while leaving anterograde transport intact [22], with an IC_50_ of ~10 nM. As illustrated in Figure 9, Mycalolide B decreased the number of neurites/SG explant by >90 percent at 3 nM, 30 nM (ANOVA, *p* < 0.01), and 300 nM (ANOVA, *p* < 0.001) (Figure 9B). The average length of neurites was reduced by >70 percent at 3 nM (ANOVA, *p* < 0.01) and >90 percent at 30 and 300 nM (ANOVA, *p* < 0.001) (Figure 9C).

**Figure 9:**
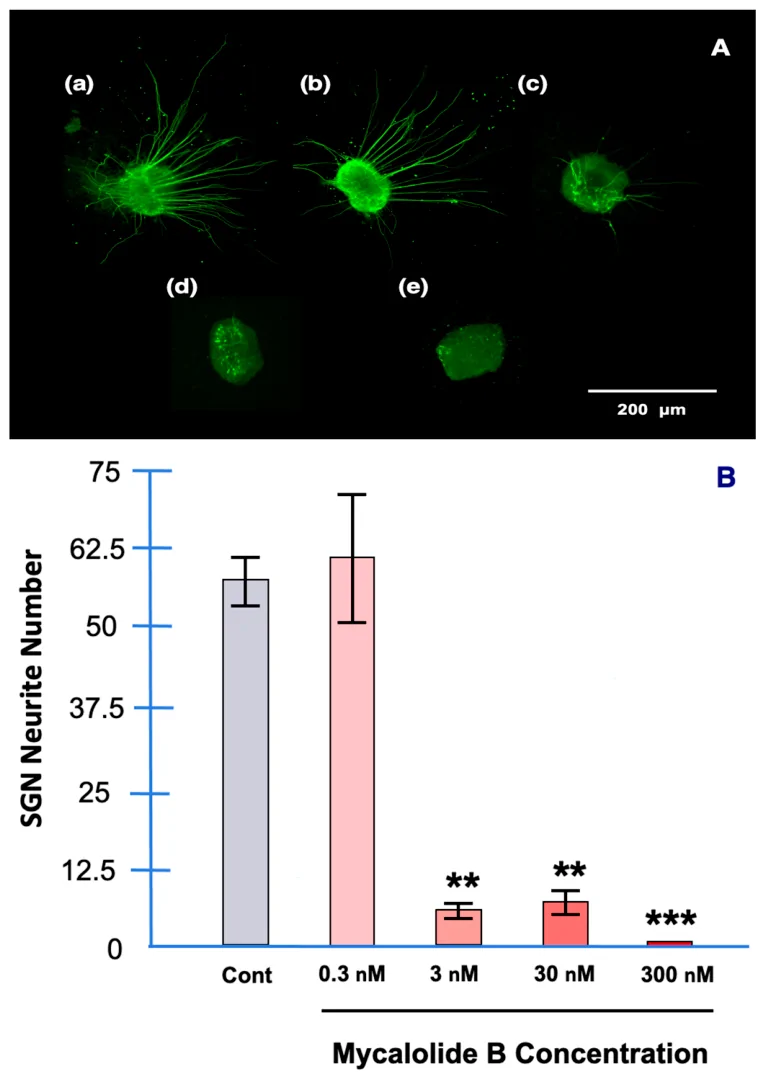

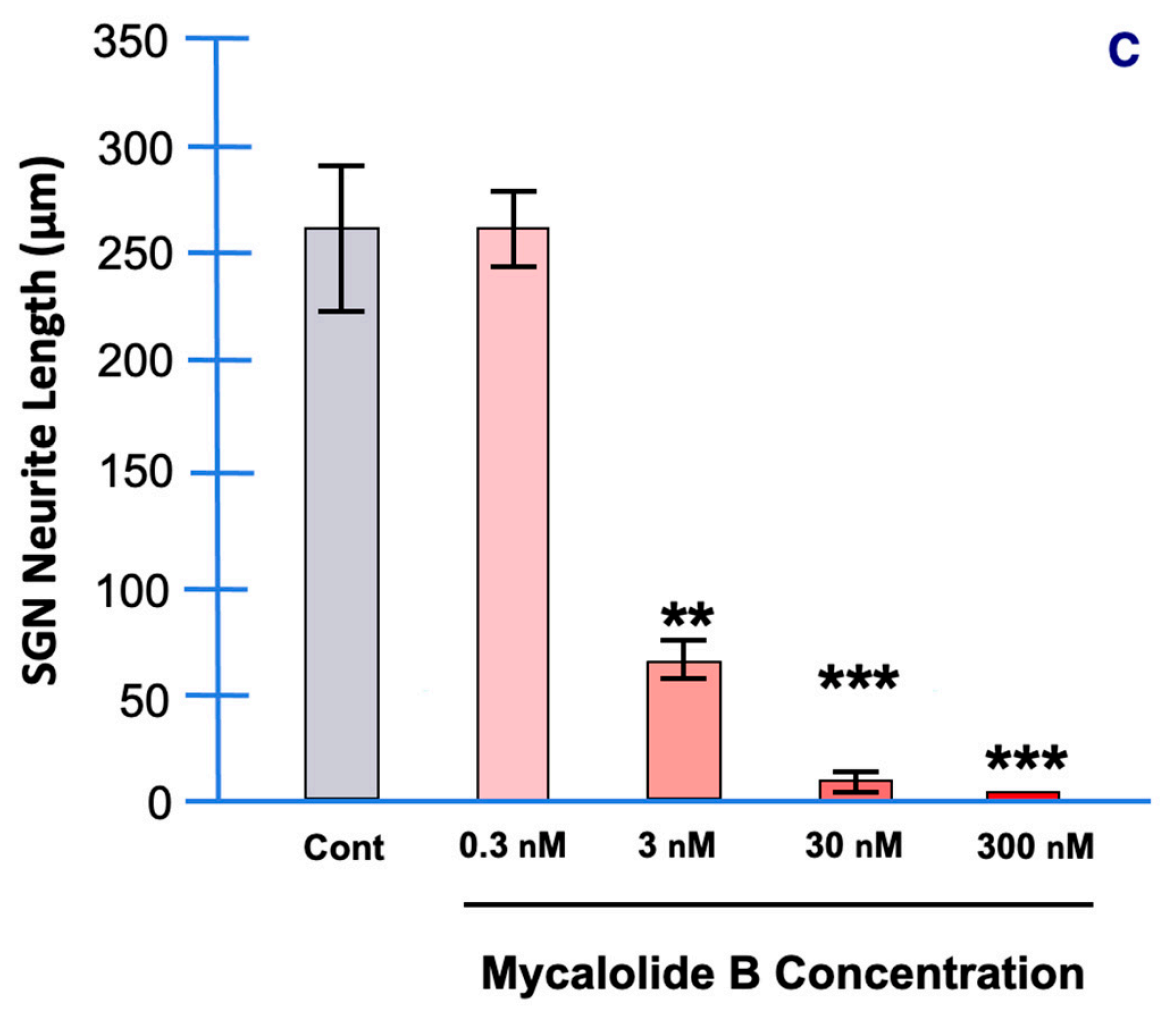
Effect of Mycalolide B, an inhibitor of retrograde neurite transport, on NT-treated SG explants. NF200 immunofluorescence. (**A**) Control explant (BDNF and NT-3 only, (**a**)) and SG explants treated with 0.3 nM; 3 nM; 30 nM; or 300 nM of Mycalolide B in the presence of BDNF and NT-3 (**b**–**e**). (**B**) Average number and (**C**) average length of neurites present on SG explants in the presence of BDNF and NT-3, as well as increasing concentrations of the Mycalolide B inhibitor. Each bar represents the average of nine explants from three independent replicates, with three explants in each replicate. ** = *p* < 0.01; *** = *p* < 0.001 (ANOVA).

## 3. Discussion

The results of this study support a role for endocytosis and endocytic signaling in the regulation of SG neurite number and length by NTs. Several inhibitors of clathrin-mediated endocytosis reduced or eliminated SGN neuritogenesis. In contrast, several inhibitors of clathrin-independent endocytosis had no effect on neuritogenesis. These data strongly implicate clathrin-dependent endocytosis in the effects of NTs. In contrast, when NTs were not present in the culture media, inhibitors of both clathrin-mediated and clathrin-independent endocytosis reduced neuritogenesis. This indicates that neuritogenesis in the absence of exogenous NTs is more complex. In contrast, the inhibitor of retrograde transport into Golgi enhanced neuritogenesis, indicating a role for this pathway in receptor removal from the growth cone, and potentially in nuclear signaling by receptor- and NT-containing endosomes.

Our results also provide evidence regarding the endocytic mechanism involved. Dynasore, a dynamin inhibitor of both clathrin-dependent and most forms of clathrin-independent endocytosis, nearly eliminated neurite outgrowth induced by combined treatment with BNDF and NT-3, leaving only a small fraction of neurite growth. Since both NTs are known to be independently effective at stimulating neuritogenesis in neonatal rat cochlear SG explants [23], this result provides robust evidence that endocytosis is not only involved in SGN signaling by these NTs and their cognate receptors, TrkB and TrkC, but is also required for effective Trk signaling. It should be noted that NT-3 is also known to be a low-affinity ligand for TrkA receptors; however, TrkA and its high-affinity ligand, nerve growth factor, do not drive NT signaling in the cochlea [24]. It should also be noted that in addition to endocytosis inhibition, Dynasore induces actin depolymerization, which could affect neurite outgrowth. The effects of actin cytoskeleton perturbation on endocytosis could be very indirect (e.g., changes in membrane tension, receptor clustering, and lateral diffusion or membrane fluidity). However, many studies in other systems have shown that induction of actin depolymerization has a biphasic effect on neurite outgrowth, in a push–pull manner [18,25,26,27,28,29]. We did not observe a biphasic response with Dynasore or any of our other inhibitors, suggesting that actin depolymerization was not responsible for the observed effects of Dynasore. Instead, cytochalasin-B, which is also an inhibitor of actin polymerization, produced concentration-dependent suppression of neurite number and length, indicating that actin polymerization is required for SGN neuritogenesis under both basal and neurotrophin-stimulated conditions.

Thus, the effect of Dynasore should be interpreted cautiously, because dynamin inhibition may suppress neurite outgrowth through impaired Trk-containing endosome formation as well as through effects on actin-dependent growth cone dynamics. However, the convergent inhibition observed with Dynasore, chlorpromazine, and ES9-17 supports a major role for clathrin/dynamin-mediated internalization in neurotrophin-dependent SGN neuritogenesis.

Pitstop 2 is an inhibitor of clathrin, which acts by interacting at several sites on the molecule [30] to block clathrin-mediated endocytosis. Treatment with the highest dosage of Pitstop 2 reduced SG explant neurite growth by more than 90% (Figure 6). Pitstop 2 has been reported to block clathrin-independent endocytosis. However, treatment with Filipin, which blocks most forms of clathrin-independent endocytosis, produced no significant changes in neurite number or extension. This finding indicates that endocytosis of NT receptors in SGNs is primarily by clathrin-dependent endocytosis.

It should be noted that the number of known clathrin-independent endocytic mechanisms continues to increase, including some that do not involve lipid rafts, and Filipin does not inhibit all forms [8]. Also, based on published IC_50_ data (see Table in the methods), dynamin inhibition in our study may have been more effective in inhibiting SGN neurite number and length than clathrin inhibition. It is thus possible that Filipin-resistant, clathrin-independent endocytosis also plays a role in SG NT signaling. This could include macroendocytosis [9]. Unfortunately, no specific pharmacological inhibitor of macroendocytosis has been identified [31], and this possibility was not tested.

In contrast to endocytosis inhibition, an inhibitor of retrograde transport of endosomes into the trans-Golgi network, Retro-2, with an IC_50_ of 5 µM [21], significantly increased the length of neurites on each SG explant compared to the controls at 30 µM (Figure 8, ANOVA, *p* < 0.05), and had no effect on neurite number. This finding suggests that the Golgi network may play a significant role in the degradation of receptors that otherwise might be recycled to the cell membrane from early endosomes [10].

The dramatic reduction in neurite number and length by treatment with a selective inhibitor of retrograde neurite transport, Mycalolide B, provides robust evidence that retrograde transport of signaling endosomes can regulate SG neurite formation and extension. This finding supports the role of signaling endosomes and their transport to the SGN soma to potentially regulate gene expression and associated protein translation central to neuritogenesis and growth. Retrograde transport may also divert endosomes that could be recycled to the growth cone membrane. While Mycalolide B also induces actin depolymerization, which could affect neurite outgrowth, we did not observe a bipartite response for neurite length or number from low to high dosages. This suggests that inhibition of retrograde neurite transport most likely underlies the effects of Mycalolide B. Both BDNF/TrkB and NT-3/TrkC could be present in retrogradely transported signaling endosomes. However, in other systems, differences in transport of signaling endosomes have been noted, depending upon the type of NT receptor [6,12,32]. A separate examination of the trafficking of these NTs could reveal differences in SG neurons.

Of course, any study that utilizes inhibitors to assess mechanisms must be interpreted with caution. Most inhibitors have effects on other processes than their intended targets, especially at higher dosages, and this is equally true of endocytosis inhibitors [31]. For this reason, we employed multiple concentrations, chosen to bracket the generally accepted IC_50_. Moreover, it would appear that most of the off-target inhibitor effects are inconsistent with our results. For example, in addition to dynamin inhibition, Dynasore has been found to reduce labile cholesterol in the plasma membrane and disrupt lipid raft organization [33]. However, Filipin, which sequesters cholesterol and disrupts lipid rafts as its primary mechanism of action (e.g., [34]), had no effect on SG neurite outgrowth. This indicates that Dynasore’s lipid raft activity is unlikely to have accounted for its effects on SG neurons. Similarly, at sufficiently high concentrations, Filipin can inhibit clathrin-mediated endocytosis. However, we used dosages that limit activity to clathrin-independent endocytosis. As noted above, both Dynasore and Mycalolide B can cause actin depolymerization, which could disrupt neurite growth independent of endocytosis and retrograde transport. Notably, cholesterol perturbation itself has been shown to alter actin filament assembly [35], further suggesting that any indirect cytoskeletal effects of our inhibitors would likely have manifested in our Filipin-treated cultures. However, actin depolymerization has been shown in many studies to have a biphasic effect on neurite extension. Low dosages of actin depolymerization agents increase neurite growth, while higher dosages inhibit growth [18,25,27,28,29]. We did not observe a bipartite effect with any of our inhibitors, which argues against an actin depolymerization effect.

There are a number of limitations to our study. The inhibitor findings presented here will need to be validated using a different rat strain such as Wistar or Long-Evans, as well as through genetic gain- and loss-of-function approaches, competition assays and/or very high-resolution immunofluorescence imaging. Such studies were beyond the scope of this project, but can be the subjects of future research. Any of the inhibitors used could have as yet undiscovered off-target effects. While we strove to create explants of equal size, variation could have influenced our results. We did not measure the endogenous levels of neurotrophins produced by the SG explants themselves. Thus, our results primarily apply to the enhancement of neurite growth produced by the exogenous neurotrophins. Finally, cell and neurite viability was not assessed independently, and a contribution from treatment-related cytotoxicity cannot be excluded. We fully acknowledge that because of these limitations, some uncertainty remains regarding our results, which can only be resolved by future experiments. Previous studies have reported sustained growth of rat SG explants for up to 7 days in culture [23,36,37,38]. Our protocol with 24 h for attachment followed by 72 h of treatment, for a total of 96 h, is well within this established interval, but treatment-specific effects on viability cannot be excluded. Nonetheless, whereas earlier SGN studies established that neurotrophins and other extracellular cues regulate SGN survival, morphology, and neurite regeneration [2,15,39], our inhibitor data provide insights into endocytic trafficking and Trk-dependent signaling.

The finding that Retro-2 enhanced neurite growth has potential clinical implications. Recent research indicates that exposure of the ear to damaging stimuli, such as noise or ototoxic drugs, leads not only to the loss of HCs but also to the loss of synapses on the innermost row of HCs, responsible for transmission of most auditory information to the central auditory system, and the retraction of the associated afferent SG dendrites. This synaptopathy can occur even when in the absence of any threshold shift, resulting in what has been termed “hidden hearing loss” [40,41]. Loss of neural connections has been proposed to compromise central processing of complex acoustic signals, as well as hearing in noisy environments. Lost synapses can be restored by NT therapy (e.g., [42]). Enhancement of NT signaling by inhibition of retrograde transport into Golgi could increase the effectiveness of emerging NT therapies that target the regrowth of SG dendrites and restoration of synaptic connections with HCs. Of course, it must be noted that the present study was performed on immature SGNs in culture, whereas any treatment would need to be effective on adult SGNs in vivo. Studies in the adult cochlea of live animals would be required before conclusions regarding the use of inhibitors of retrograde transport of endosomes into the trans-Golgi network could be more definitively drawn.

## 4. Materials and Methods

### 4.1. Animals

Neonatal (3–5 days) outbred Sprague-Dawley (SD) rats were used. All experiments were performed in accordance with National Institutes of Health guidelines and were approved by the VA San Diego Medical Center IACUC (Protocol A21-007; approval expires 3 December 2027).

### 4.2. SG Micro-Dissection and Explant Culture

A set of 24-well tissue culture plates were precoated overnight at 4 °C with 10 μg/mL fibronectin from human plasma (Sigma-Aldrich, St. Louis, MO, USA). After 24 h, the wells were washed twice with phosphate-buffered saline (PBS) and incubated at 37 °C for 1 h with 300 μL of 20 μg/mL poly-L-lysine (PLL) in Dulbecco’s modified Eagle’s medium (DMEM). Wells were washed twice with PBS and filled with 170 μL of media consisting of DMEM (Gibco, Grand Island, NY, USA) with 10% fetal bovine serum (FBS, Gibco), 10 mM HEPES buffer (Gibco), and 300 U/mL penicillin (Sigma-Aldrich), to promote explant attachment, for 24 h. Thereafter, FBS was not used.

The SG was harvested from the cochlea of neonatal (3–6 days after birth) rats euthanized by anesthesia with rodent cocktail (2.0 mg/kg xylazine and 40.0 mg/kg ketamine i.p.) followed by decapitation. The lateral wall tissues and organ of Corti were first removed, and the ganglion separated from the modiolus in one long strip with fine forceps. The entire ganglion was then divided into eight explants, which ensured that there was no sampling bias toward any part of the ganglion. Care was taken to generate explants of approximately equal size, but some variation was unavoidable. Measurement of explant size after attachment is difficult, as explants spread in variable ways on the culture surface. Each explant was placed in the center of one well and incubated overnight at 37 °C with 5% CO_2_.

After attachment, explants were cultured in 200 μL of maintenance medium composed of DMEM supplemented with 10 μL/mL N2 supplement-A, 10 mM HEPES buffer, 300 U/mL penicillin, 5 mg/mL glucose, and 0.5% DMSO (all from Invitrogen/Gibco). To promote neurite outgrowth, the medium also contained 10 ng/mL BDNF (Calbiochem, La Jolla, CA, USA) and 25 ng/mL NT-3 (MilliporeSigma, St Louis, MO, USA). Individual inhibitors (concentrations listed in Table 1), solubilized in 0.5% DMSO, were added as indicated. Cultures were maintained at 37 °C in 5% CO_2_ for 72 h. Control explants were treated with neurotrophins and 0.5% DMSO only. All experiments were performed as three independent biological replicates. Neurite viability was not assessed, but prior studies have shown that SG neurites continue to grow for up to 10 days in the culture conditions employed for this study [23,36,37,38]. Our culture interval of 3 days after 24 h for attachment is well within this interval.

Cochlear spiral ganglion (SG) neurons are bipolar, with peripheral neurites serving as dendrites and central axons extending from their somata in Rosenthal’s canal to the cochlear nucleus via the VIIIth cranial nerve. In our neonatal rat cochlear explant model, the neurites observed predominantly represent dendrites, as axonal extension typically requires specialized dissection procedures. Whitlon et al., [49] reported that certain culture conditions, including the addition of LIF to the medium, which we did not employ, can enhance bipolar neurite outgrowth in SG neuron cultures. In rats, Type I and Type II SG neurons cannot be distinguished in culture. By contrast, in mice, peripherin expression is restricted to Type II neurons, and at the developmental stage examined here, most neurites originate from Type I neurons [50,51].

### 4.3. Immunohistochemistry

At the end of 72 h culture, SG explants were fixed by immersion in 4% paraformaldehyde in 0.1 M phosphate buffer, pH 7.2, for 30 min at room temperature (RT). After three washes with PBS, the tissues were incubated with PBS containing 1% glycine and blocking solution (PBS containing 4% FBS and 0.2% Triton X-100) for 30 min each at RT to reduce nonspecific antibody binding. The explants were incubated with rabbit anti-neurofilament 200 antibody (Sigma, 1:500) at 4 °C overnight. The next day, after two washes in PBS, the tissues were incubated with FITC-conjugated anti-rabbit IgG secondary antibody (Jackson ImmunoResearch, 1:100) for 1 h at RT. After two washes in PBS, the tissues were maintained in PBS at 4 °C until imaging using the FSX100 microscope (Olympus, Tokyo, Japan).

### 4.4. Data Analysis

The immunostained SG explants were mounted in Vectamount (Vector Labs) under a coverslip and imaged by epifluorescence microscopy. All neurites extending from each explant were traced using ImageJ (version 1.5), and the number and average neurite length were quantified. Length was measured to the most distal neurite tip, excluding peripheral branches. Responses of explants to BDNF and NT-3 varied with different batches of factors. An inhibitor was always applied in combination with the same factors used for control explants. Two independent observers performed the evaluations, blind to the treatment of each explant. Inter-observer variability was less than 5%, and the two measurements were averaged for each explant. Statistical analyses were conducted in StatView (version 5.0) using one-way ANOVA. Data were first tested for normal distribution, and pairwise post hoc comparisons with Bonferroni correction were applied to assess treatment group differences at α = 0.05.

## 5. Conclusions

The inner hair cell–spiral ganglion synapse is among the most metabolically active ribbon synapses in the nervous system, characterized by high vesicle turnover and sustained neurotransmitter release [52]. The results of this study shed significant light on the processes involved in stimulation of SGN processes by NTs. They identify clathrin/dynamin-mediated internalization as the primary mechanism of Trk endocytosis into SG neurons. They determine that receptor endocytosis is required for NT-mediated generation of SGN neurites and their extension. They demonstrate that the retrograde transport of signaling endosomes along neurites to the SGN soma affects neurite generation and extension. In addition, they indicate a role for the trans-Golgi network in the degradation of NT receptors in SGNs. The latter finding suggests a potential means by which to increase neurite extension in response to NT therapy for cochlear synaptopathy.

## Figures and Tables

**Figure 1:**
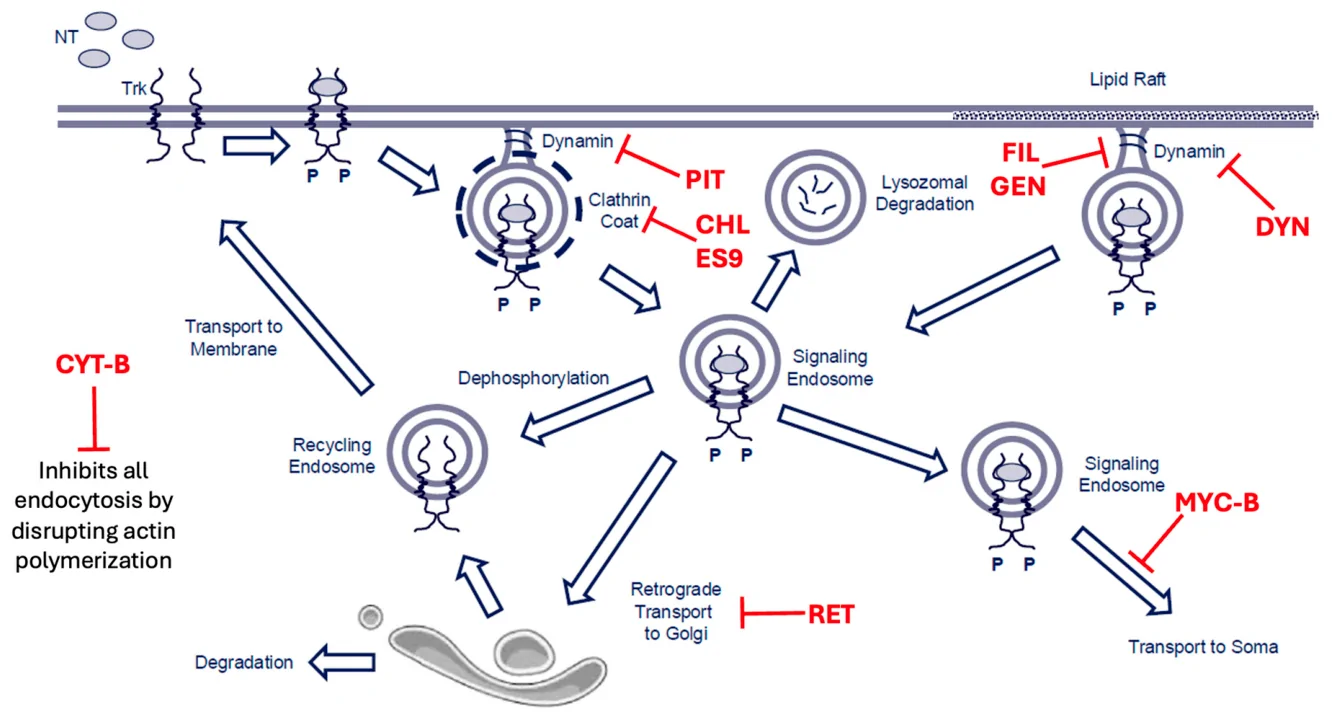
Trk receptor internalization. Endocytic internalization of transmembrane tyrosine kinase (Trk) receptors following binding of neurotrophins (NTs). In this illustration, NT binding triggers dimerization of the Trk receptors, preceding endocytosis that can be initiated by clathrin and dynamin. Alternatively, after Trk receptors incorporate into lipid rafts, endocytosis can be mediated by dynamin alone. A third potential endocytic mechanism, macroendocytosis, independent of both clathrin and dynamin, is not illustrated. After internalization, the receptors within the “signaling endosomes” may be degraded, recycled directly to the membrane, transported into the Golgi apparatus, or transported to the soma by retrograde neurite transport to regulate gene expression. Once incorporated into the Golgi, receptors can be degraded or repacked into recycling endosomes and transported back to the cell membrane (adapted from [6]). The inhibitors used in this study and their targets are indicated in red. See Table in methods for abbreviations.

**Figure 2:**
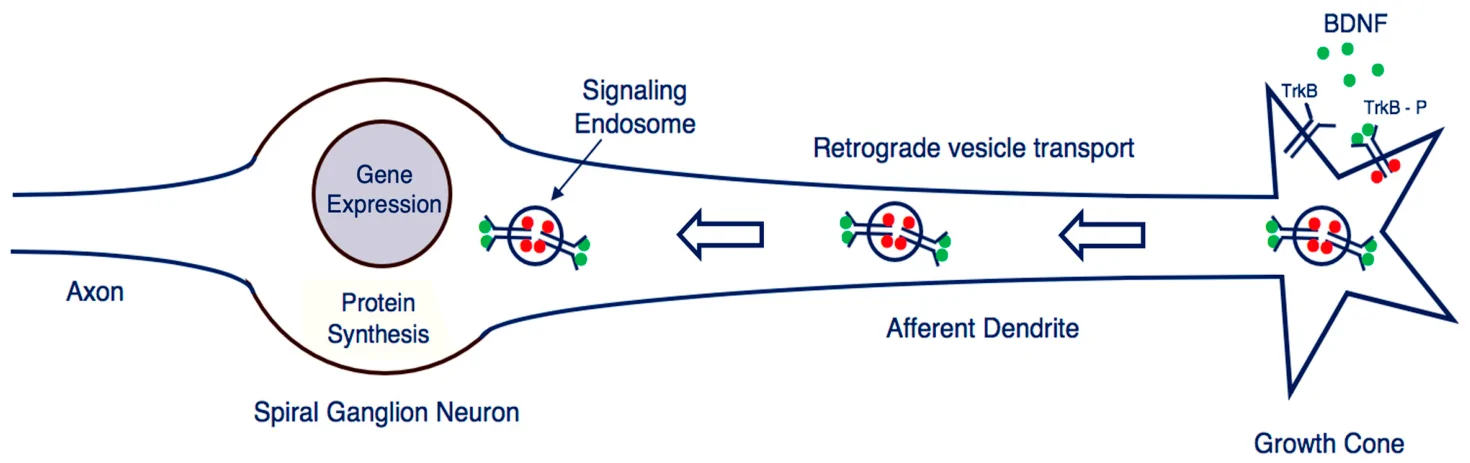
Retrograde transport of signaling endosomes. After neurotrophin (BDNF in this example) interacts with Trk receptors at the growth cone of a neurite, inducing autophosphorylation, the receptors are internalized. Retrograde transport can carry signaling endosomes to the cell body.

**Table 1:** Inhibitors used to interrogate neurotrophin signaling pathways. Inhibitors, concentrations evaluated, and primary mechanisms of action are listed. Compounds selectively target clathrin-mediated endocytosis, clathrin-independent/lipid raft-dependent uptake, dynamin-dependent vesicle scission, and retrograde intracellular trafficking. Dosages span those used in other studies of endocytosis.

Inhibitor	Concentrations	Mechanism	Supplier, ≥97.5% Purity by HPLC
Chlorpromazine (CHL)	3, 15, 30, 60 μM	Inhibits caveolin-dependent endocytosis [43]	Sigma-Aldrich
Cytochalasin-B (CYT-B)	0.2, 2, 20, 200 nM	Inhibits actin polymerization and phagocytosis [44,45]	Sigma-Aldrich
Dynasore (DYN)	15, 25, 50, 75 μM	Inhibits clathrin-mediated and most clathrin-independent endocytosis [17,33]	Sigma-Aldrich
ES9-17 (ES9)	1, 10, 50, 100 μM	Endosidin 9 analog that inhibits clathrin-mediated endocytosis targeting the clathrin heavy chain (CHC) [16]	Biosynth- Carbosynth
Filipin (FIL)	1, 5, 10, 25 μg/mL	Inhibits most forms of clathrin-independent endocytosis [20,34,35]	Sigma-Aldrich
Genistein GEN	2, 10, 20, 40 μM	Inhibits clathrin-independent endocytosis based on lipid rafts and caveolae [46,47,48]	Sigma-Aldrich
Mycalolide B MYC-B	0.3, 3, 30, 300 nM	Inhibits retrograde transport along neurites [22]	Enzo
Pitstop 2 PIT	5, 7.5, 10, 25 μM	Inhibits clathrin-mediated endocytosis [19]	Sigma-Aldrich
Retro-2 RET	3, 30, 300 μM	Inhibits retrograde transport into the trans-Golgi network [21]	Sigma-Aldrich

## Data Availability

The raw data supporting the conclusions of this article will be made available by the authors on request. Please email afryan@health.ucsd.edu OR akurabi@health.ucsd.edu.

## Abbreviations

The following abbreviations are used in this manuscript:

ANOVA	Analysis of variance
BDNF	Brain-derived neurotrophic factor
DMSO	Dimethyl sulfoxide
DMEM	Dulbecco’s modified Eagle medium
h	Hour
HC	Hair cell
HEPES	4-(2-hydroxyethyl)-1-piperazineethanesulfonic acid
IC_50_	Half maximal inhibitory concentration
NT	Neurotrophin
PLC-γ	Phospholipase C-γ
RT	Room temperature (20-22 °C)
SEM	Standard error of mean
SG	Spiral ganglion
SGNs	Spiral ganglion neurons
Trk	Tropomyosin receptor kinase
TRPC	Canonical transient receptor potential channel
